# Sensory Abnormality and Quantitative Autism Traits in Children With and Without Autism Spectrum Disorder in an Epidemiological Population

**DOI:** 10.1007/s10803-019-04237-0

**Published:** 2019-10-03

**Authors:** K. Jussila, M. Junttila, M. Kielinen, H. Ebeling, L. Joskitt, I. Moilanen, M.-L. Mattila

**Affiliations:** 1 Clinic of Child Psychiatry, PEDEGO Research Unit, University of Oulu and Oulu University Hospital, P.O.B. 26, 90029 Oulu, Finland; 2 Faculty of Education, University of Oulu, Oulu, Finland

**Keywords:** Autism, Autism spectrum disorder, ASD, ASSQ, Sensory abnormalities, Sensory processing

## Abstract

Sensory abnormalities (SAs) are recognized features in Autism Spectrum Disorder (ASD), and a relationship between SAs and ASD traits is also suggested in general population. Our aims were to estimate the prevalence of SAs in three different settings, and to study the association between SAs and quantitative autism traits (QAT) using the Autism Spectrum Screening Questionnaire (ASSQ) and a parental questionnaire. In an epidemiological population of 8-year-old children (n = 4397), the prevalence of SAs was 8.3%, in an ASD sample (n = 28), 53.6%, and in a non-ASD sample (n = 4369), 8.0%, respectively. Tactile and auditory hypersensitivity predicted an ASD diagnosis. The ASSQ was able to differentiate children with and without SA. In conclusion, QAT level and SAs were associated in all study samples.

Autism Spectrum Disorder (ASD) is a pervasive neuropsychiatric disorder characterized by deficits in two main domains: limitations in social communication and interaction, and stereotyped, repetitive patterns of behavior (APA 2013). The prevalence of ASD is estimated to be 0.6–0.8% (Fombonne 2009), but higher prevalences have also been published, e.g., 1.34% (Christensen et al. 2016) and 2.64% (Kim et al. 2011). In Finland, the prevalence of ASD is 0.84% (Mattila et al. 2011). However, instead of the traditional consideration of ASD as a clinical condition distinct from the general population, recent research recognizes ASD as the severe end of a continuum, where autistic traits are continuously distributed across the population (Constantino and Charman 2016; Constantino and Todd 2003; Posserud et al. 2006).

Autism spectrum disorder manifests as a wide variability of impairments in reciprocal social behavior (RSB) and these social, communication and cognitive difficulties are considered the core deficiency of ASD. The term RSB refers “to the extent to which an individual can engage in emotionally appropriate, turn-taking social interaction with others. RSB requires the individual to be cognizant of the emotional and interpersonal cues of others, to appropriately interpret and respond to those cues, to be aware of others’ perceptions or restrictions to his or her own behaviors, and to be capable of emotional engagement” (Constantino et al. 2000).

Repetitive behavior consists of five subcategories: repetitive sensory-motor/stereotypic behaviors, ritualistic/insistence on sameness behaviors, compulsive behavior, restricted/circumscribed interests and self-injurious behaviors (Bishop et al. 2013). These repetitive behaviors are more likely among children with ASD who also experience sensory abnormalities (SAs) (Chen et al. 2009; Gabriels et al. 2008), and they might function as a soothing or stimulating mechanism for children with sensory dysfunction (Leekam et al. 2011).

In addition to communication deficits and restricted repetitive patterns, the diagnostic criteria of ASD now also include SAs, that can include hyper- and hyporeactivity to sensory stimuli or unusual interest in sensory aspects of the environment (APA American Psychiatric Association 2013). Before the inclusion of atypical sensory functioning in DSM-5 as one of key symptoms of ASD, abnormalities regarding sensory stimuli had been widely observed to appear with ASD ever since Kanner’s first characterization (Kanner 1943). These abnormalities seem to persist across age: sensory symptoms of toddlers have been found to last through adulthood (Harrison and Hare 2004; Leekam et al. 2007; Minshew and Hobson 2008; Rogers et al. 2003).

The role of sensory perception in autism is not yet fully understood, but the recent research has acknowledged it as another core feature of ASD, possibly the most primal one. In a review article published in Nature Reviews, the sensory symptoms are recognized as the earliest, primary characteristics of autism which predict and explain deficits in later social communication (Robertson and Baron-Cohen 2017). These atypical sensory symptoms can cause avoidance of social stimuli and thereby impact the development of social and cognitive abilities (Ben-Sasson et al. 2007).

Only a few studies have addressed the association between quantitatively assessed autistic traits and atypical sensory functioning. In all these studies, they have shown to progress in line with an increase of autistic traits in adult populations, which included both neurotypical participants and participants with ASD (Horder et al. 2014; Mayer 2017; Robertson and Simmons 2013; Takayama et al. 2014; Tavassoli et al. 2014). The association between QAT and sensory functioning in children has been studied in some studies. Hilton et al (2007) found, that severity of sensory dysfunction was positively associated with QAT (measured by the Social Responsiveness Scale) in 6–10 year-old high-functioning children with ASD. Also Adamson et al. (2006) found a moderate positive correlation between Gilliam Autism Rating Scale scores and the Short Sensory Profile scores among a group of 44 children with ASD. To our knowledge, no studies have concentrated in investigating the relationship between SAs and QAT in normative child groups.

The aims of the present study were: (1) to estimate the prevalence of SAs in three child samples: in an epidemiological child population, in an ASD child sample and in a non-ASD child sample, (2) to assess whether SAs are indicators for an elevated risk of ASD, and (3) to assess whether specific forms of SAs are associated with QAT (as measured by the ASSQ) in general child population. Based on previous literature, we hypothesized that the prevalence of SAs would be significantly higher among children with ASD than in the general child population, and that children exhibiting SAs would be more prone to subclinical QAT and/or ASD (or autistic-like behavior/phenotype).

## Methods

### Procedure and Participants

Data collection was conducted in the Northern Ostrobothnia Hospital District (NOHD) area in the Province of Oulu, Finland.

Prior to data collection, the study was approved by the Ethics Committee of the Faculty of Medicine, University of Oulu, and the Ethics Committee of the NOHD. Approval was obtained also from the school inspector, and the superintendents of 43 municipalities and 329 school principals were informed and permission was requested to collect data.

The target population of the epidemiological study included all 8-year-old children born in 1992 and living in the NOHD area during autumn 2000 (n = 5484). No exclusion criteria were used in the invitation phase. The Finnish population was homogeneous at the time of our study, mainly of Finnish extraction and Finno-Ugric origin. The children and their parents were invited to participate through schools (329 schools), of which 321 schools agreed to participate (5319 children). Of these, nine schools had no pupils born in 1992, and eight schools did not return the study material. Finally, 304 schools with 5242 (96%) children participated. The teachers of these children were given an informative lecture, after which the research material was handed out to the teachers, who distributed the material to parents. The parents were asked to complete the Autism Spectrum screening Questionnaire (ASSQ) and a developmental questionnaire, in which sensory-perceptual problems were inquired about. Parents of 4424 (84%) children gave written informed consent to participate. Since the ASSQ has been validated for children with a full-scale intelligence quotient (FSIQ) equally to or above 50 (Ehlers et al. 1999), only children with normal cognitive level or mild mental retardation were included in the study sample. Eight children were reported to have mental retardation, with a FSIQ below 50, and they were not included in further analyses.

Children meeting the suggested Swedish “high-risk” (n = 73) or the “medium-risk” (n = 52) cut-off score for screening on the ASSQ (Ehlers et al. 1999; Kadesjö et al. 1999) were invited to diagnostic evaluations (n = 125). The ADI-R and ADOS (n = 110; 88%) were administered by a pediatrician. Neurocognitive evaluations (WISC-III) of the children were performed by two psychologists to ensure that none of the children had a FSIQ below 50. School day observations of 24 children were implemented by the Master of Education graduate in order to have more information for diagnostic evaluations. Previous hospital records of the 110 screened and evaluated children were studied. ASD diagnoses (n = 26) were then defined in detail according to DSM-IV (APA 1994) based on consensus between the experienced pediatrician and a child psychiatrist based on all gathered data (ADI-R, ADOS tapes, WISC-III, school day observations, hospital register data). In addition, two screened children, who did not participate in the diagnostic evaluations in our study, had ASD diagnoses in their hospital records, and according to the developmental questionnaire filled by parents. Finally, the ASD sample consisted of 28 children with ASD.

Of children with complete information about their SAs (4397 children; 2167 boys, 2230 girls), 3565 returned the parental ASSQ, 4382 the teacher ASSQ, and 3534 returned both ASSQs. When analyzing the ASSQ scores, we used the combined parent-teacher summed total scores (Mattila et al. 2012).

## Measures

### The Autism Spectrum Screening Questionnaire

“The Autism Spectrum Screening Questionnaire (ASSQ; Ehlers et al. 1999) is a 27-item parent-/teacher-screening inventory, designed to screen ASD in children with a full-scale IQ 50 or more. It covers the main behavior areas of ASD (i.e., social interaction, communication, and restricted and repetitive behavior) as well as motor deficits/behaviors (e.g., clumsiness), and other associated symptoms such as motor and vocal tics. Items are rated on a 3-point Likert-type scale (i.e., 0 = normal, 1 = some abnormality, and 2 = definite abnormality) with total scores ranging from 0 to 54, with higher scores indicating more severe levels of social impairment. In the original validation study of the Swedish version, a cut-off score of 22 for teachers’ ratings and 19 for parental ratings was suggested for 6–17 year-old children with FSIQ at or above 50 (Ehlers et al. 1999). For Finnish primary school-aged, 7- to 12-year-old children with an FSIQ ≥ 50, the optimal cut-off score is 30 in clinical settings and 28 in total population screening using summed ASSQ scores of parents’ and teachers’ ratings (Mattila et al. 2012).

The ASSQ was developed in Sweden to be used with children aged 7–16. It is one of the most widely used autism screening instruments, and has been used in epidemiological studies e.g. in Sweden, Norway, Estonia, Denmark, China and South Wales. In the beginning of this study, the ASSQ was translated from Swedish into Finnish by two clinical psychologists and then it was back-translated into Swedish by an official Swedish–Finnish translator, and after comparison of the original Swedish and the back-translated Swedish forms, the final Finnish version was completed. For the Finnish version, the ASSQ rating expression of two points (Swedish definition “stämmer absolut”, meaning “fits definitely”), was toned down to “fits” because our clinical experience suggested that Finnish parents are reluctant to assess their children’s features as “definite”. The Finnish expression “fits” was also considered analogous to the English ASSQ rating expression of two points (“yes”) (Mattila et al. 2012).

### The Autism Diagnostic Interview-Revised

The Autism Diagnostic Interview-Revised (ADI-R; Lord et al. 1994) is a standardized investigator-based, structured parental interview developed to elicit a full range of the information needed when evaluating the diagnostic criteria of ASD. It covers the main symptom areas associated with ASD: reciprocal social interaction, communication and restricted and stereotyped behavior and interests (DSM-IV; APA 1994).

### Autism Diagnostic Observation Schedule

The Autism Diagnostic Observation Schedule (ADOS; Lord et al. 2000) is a semi-structured assessment of social interaction, communication, and play or imaginative use of materials. It comprises four modules based on the verbal level of the subject being evaluated.

Both the ADI-R and ADOS use diagnostic algorithms based on separate thresholds for the ASD symptom domains. Domain scores are sums of codings that indicate the severity of impairment based on symptom frequency and degree of interference with daily living.

The physicians (pediatrician and child psychiatrist) and Master of Education graduate who participated in the diagnostic process had been trained in the use of the ADI-R and ADOS for research purposes, but inter-rater reliabilities had not been established. The ADI-R and ADOS were not used to make diagnostic classifications in the present study (i.e., the diagnostic algorithms were not used). Instead, these instruments were used to obtain structured information from parents and for semi-structured observation of a child. A clinical best estimate was used to make the diagnosis.

### The Wechsler Intelligence Scale for Children-Third Revision

The Wechsler Intelligence Scale for Children, 3rd. ed. (WISC-III; Wechsler 1991) is a performance scale designed for children ages 6–16. It consists of verbal and visual performance subtests. Verbal subtests include (1) information (factual knowledge, long-term memory, recall), (2) similarities (abstract reasoning, verbal categories and concepts), (3) arithmetic (attention and concentration, numerical reasoning), (4) vocabulary (language development, word knowledge, verbal fluency), (5) comprehension (social and practical judgment, common sense), and (6) digit span (short-term auditory memory, concentration). The visual performance subtests include (1) picture completion (alertness to detail, visual discrimination), (2) coding (visual-motor coordination, speed, concentration), (3) picture arrangement (planning, logical thinking, social knowledge), (4) block design (spatial analysis, abstract visual problem-solving), (5) object assembly (visual analysis and construction of objects), (6) symbol search (visual-motor quickness, concentration, persistence), and (7) mazes (fine motor coordination, planning, following directions).

### The Developmental and Background Questionnaire

A 14-item parental questionnaire was used to gather information about the participants’ early development and familial background. The questionnaire assessed sensory hyper- and hyposensitivity as follows: (1) “Does the child have sensory hypersensitivity in the area of one or more sensory modalities: auditory, olfactory, gustatory, tactile or visual?”, and (2) “Does the child have sensory hyposensitivity in the area of one or more sensory modalities: auditory, olfactory, gustatory, tactile or visual?”

### Statistical Methods

Analyses were performed with the Statistical Package for Social Sciences (IBM SPSS Statistics v. 24).

To investigate the association between SAs and ASD, a series of logistic regression analyses was used as a risk analysis.

To investigate the association between SA and QAT, we compared the ASSQ scores in samples with and without SA by using the non-parametric Mann–Whitney and Kruskal–Wallis tests for two and three independent samples since the ASSQ scores were not normally distributed. To avoid possible misleading influences of sample size, we estimated effect size (ŋ^2^) in addition to determining p-values. Effect size describes the proportion of variability explained by a given variable of the variance remaining after excluding variance explained by other predictors; it quantifies the effect of an independent variable (here, the SAs) to the variation in the dependent variable (here, the ASSQ scores), thus describing the observed effect instead of merely identifying a statistical significance (Fritz et al. 2012). For eta square (ŋ^2^), the effect size is considered to be small, when ŋ^2^ > .01, moderate, when ŋ^2^ > .06, and large, when ŋ^2^ > .14.

## Results

Of the 4397 children with sufficient sensory data, 8.3% (n = 364; 206 males and 158 females) were reported to have some form of sensory-perceptual abnormality. Among the children with ASD (n = 28), the prevalence of SA was 53.6%, (n = 15, 11 males, 4 females), and among the non-ASD children 8.0% (n = 349; 195 males, 154 females), respectively (Table 1).

**Table 1:** Prevalence (percentage) of sensory abnormalities (parental report)

	ASD sample N = 28 (%)	Males N = 17 (%)	Females N = 11 (%)	Non-ASD sample N = 4369 (%)	Males N = 2150 (%)	Females N = 2219 (%)
Any sensory abnormality	53.6	64.7	36.4	8.0	9.1	6.9
Hypersensitivity						
Auditory	42.9	47.1	36.4	3.3	4.1	2.5
Visual	0	0	0	0.4	0.4	0.3
Tactile	17.9	23.5	9.1	0.6	0.8	0.5
Gustatory	7.1	11.8	0	0.9	1.2	0.7
Olfactory	25.0	29.4	18.2	1.5	1.7	1.3
Hyposensitivity						
Auditory	0	0	0	1.4	1.7	1.0
Visual	0	0	0	1.4	1.3	1.4
Tactile	0	0	0	0.1	0.1	0.1
Gustatory	0	0	0	0	0	0
Olfactory	0	0	0	0.1	0	0.1

*ASD* autism spectrum disorder

A series of logistic regression analyses revealed that the presence of any form of SA indicated a 13-fold risk for ASD diagnosis (OR 13.3, 95% Confidence Interval [CI] 6.3–28.2, p < 0.001). Tactile hypersensitivity raised the risk to a 34-fold (OR 33.7, 95% CI 12.0–95.0, p < 0.001), and auditory hypersensitivity to a 22-fold (OR 22.0, 95% CI 10.2–47.4, p < 0.001). For more specific risk estimates see Table 2.

**Table 2:** Risk estimate of SA for ASD diagnosis

	N = 4397			
	ASD yes/no	p Value	OR	95% CI
Any sensory abnormality				
No	13/4020		1	
Yes	15/349	< 0.001	13.3	6.3, 28.2
Auditory hypersensitivity				
No	16/4225		1	
Yes	12/144	< 0.001	22.0	10.2, 47.4
Tactile hypersensitivity				
No	23/4341		1	
Yes	5/28	< 0.001	33.7	12.0, 95.0
Olfactory hypersensitivity				
No	21/4303		1	
Yes	7/66	< 0.001	21.7	8.9, 52.9
Gustatory hypersensitivity				
No	26/4329		1	
Yes	2/40	0.005	8.3	1.9, 36.3
Visual hypersensitivity			1	
No	28/4353			
Yes	0/16	0.999	0.0	0.0, –

*SA* sensory abnormality, *ASD* autism spectrum disorder, *OR* odds ratio, *CI* confidence interval

The ASSQ (summed parents’ and teacher’s score) was able to differentiate the samples with (n = 298) and without (n = 3236) SAs statistically significantly (M = 9.4 ± 12.4 vs. 3.1 ± 5.2, p < 0.001, respectively).

When evaluating the specific SAs within the child samples, it was found that among children with ASD, the ASSQ differentiated statistically significantly only the samples with and without auditory hypersensitivity, children with auditory hypersensitivity having higher ASSQ outcome measures than those without. Auditory hypersensitivity explained 28% of the variance in the ASSQ scores among the ASD sample (M = 48.8, sd = 10.8 vs. M = 37.8, sd 7.7, p = 0.003, ŋ^2^ = 0.28), whereas in the non-ASD sample, children with hypersensitivity in any sensory modality or auditory, tactile or visual hyposensitivity had statistically significantly higher ASSQ total scores than children without. For more specific ASSQ outcome score-differences between child samples see Tables 3 and 4.

**Table 3:** Group differences of summed total ASSQ scores in child samples based on individual SAs

	ASD N = 28					Non-ASD N = 3506				
	N	M	sd	md	p/ŋ^2^	N	M	sd	md	p/ŋ^2^
Any sensory abnormality										
No	13	37.4	7.8	36	0.017	3223	2.9	4.8	1	< 0.001
Yes	15	46.9	10.8	49	0.2015	283	7.4	8.7	4	0.0374
Auditory hypersensitivity										
No	16	37.8	7.7	36	0.003	3386	3.1	5	1	< 0.001
Yes	12	48.8	10.8	52	0.2824	120	9.1	9.9	5.5	0.0256
Olfactory hypersensitivity										
No	21	40.9	10.2	38	0.192	3451	3.3	5.3	1	< 0.001
Yes	7	47.3	10.7	49	0.063	55	5.6	6.6	3	0.0042
Gustatory hypersensitivity										
No	26	42.6	10.7	40	0.737	3472	3.3	5.3	1	< 0.001
Yes	2	41	11.3	41	0.0046	34	8.2	9.5	4.5	0.0059
Tactile hypersensitivity										
No	23	41.5	10.3	39	0.351	3484	3.3	5.2	1	< 0.001
Yes	5	47	11.7	49	0.033	22	12.7	13.1	6	0.0071
Visual hypersensitivity										
No	28	42.5	10.5	40	–	3492	3.3	5.3	1	0.004
Yes	0				–	14	10.5	10.6	9.5	0.0022
Auditory hyposensitivity										
No	28	42.5	10.5	40	–	3463	3.2	5.2	1	< 0.001
Yes	0				–	43	8.1	9.6	4	0.0045
Olfactory hyposensitivity										
No	28	42.5	10.5	40	–	3504	3.3	5.3	1	0.213
Yes	0				–	2	4.5	2.1	4.5	0.0005
Gustatory hyposensitivity										
No	28	42.5	10.5	40	–	3505	3.31	5.3	1	0.508
Yes	0				–	1	0	0	0	0.0004
Tactile hyposensitivity										
No	28	42.5	10.5	40	–	3503	3.3	5.3	1	0.001
Yes	0				–	3	21	14.2	26	0.002
Visual hyposensitivity										
No	28	42.5	10.5	40	–	3455	3.3	5.3	1	< 0.001
Yes	0				–	51	6.5	7.7	3	0.0045

*ASSQ* Autism Spectrum Screening Questionnaire, *SA* sensory abnormality, *ASD* autism spectrum disorder, *ŋ*^*2*^ effect size (small > .01, moderate > .06, large > .14)

**Table 4:** Group differences of summed total ASSQ scores in child samples based on combined SAs

	ASD N = 28					Non-ASD N = 3506				
	N	M	sd	md	p/ŋ^2^	N	M	sd	md	p/ŋ^2^
Gustatory + olfactory hypersensitivity										
No	21	40.9	10.2	38	0.232	3428	3.2	5.2	1	< 0.001
Either one	5	49.8	10.6	56	0.042	67	6.1	7.9	3	0.007
Both	2	41	11.3	41		11	8.1	8	5	
Auditory + gustatory hypersensitivity										
No	14	37.3	7.5	36	0.008	3357	3.1	4.9	1	< 0.001
Either one	14	47.6	10.8	50.5	0.19	144	8.5	9.2	5	0.029
Both	0					5	15.2	15.7	9	
Auditory + olfactory hypersensitivity										
No	13	37.4	7.8	36	0.014	3339	3.1	5	1	< 0.001
Either one	11	44.6	11	43	0.229	159	7.8	9.3	4	0.026
Both	4	53.3	8.4	56		8	10.5	7.2	10	
Tactile + auditory hypersensitivity										
No	14	37.3	7.5	36	0.018	3368	3.1	4.9	1	< 0.001
Either one	11	46.7	10.8	49	0.206	134	8.7	9.4	5.5	0.03
Both	3	51	12.3	56		4	26	15.5	28.5	
Tactile + olfactory hypersensitivity										
No	20	41	10.5	38.5	0.437	3431	3.2	5.2	1	< 0.001
Either one	4	42.5	9.3	39	0.011	73	8	9.6	3	0.01
Both	4	49.5	11.9	52.5		2	2	1.4	2	
Tactile + gustatory hypersensitivity										
No	23	41.5	10.3	39	0.353	3452	3.2	5.2	1	< 0.001
Either one	3	51	12.3	56	0.01	52	8.2	9.2	5	0.011
Both	2	41	11.3	41		2	32.5	13.4	32.5	

*ASSQ* Autism Spectrum Screening Questionnaire, *SA* sensory abnormality, *ASD* autism spectrum disorder

*ŋ*^*2*^ effect size (small > .01, moderate > .06, large > .14)

## Discussion

### Prevalence of Sensory Abnormalities

To our knowledge, the present study is the first study that estimates the prevalence of SAs in an epidemiological child population yielding prevalence figures in three different settings. The prevalence of SAs was 8.3% in the total population, and 8.0 in the non-ASD sample. In population-based studies, the prevalence of sensory processing disorders in child samples has varied from 5 to 13% (Ahn et al. 2004), and sensory over-responsivity among a general elementary-school-aged child population (n = 925) even up to 16.5% concerning auditory and tactile sensations (Ben-Sasson et al. 2009).

Among children with ASD, the prevalence of SAs was 53.6% in our study. Previously in clinic-based studies, SAs have been estimated to affect even 69–95% of children with ASD (Baranek et al. 2006; Tomchek and Dunn 2007). The different results between these clinic-based studies and our epidemiological-based study are most likely explained by differences in the degree of severity of autistic symptomatology in the child samples. In clinical studies, participants with ASD are more likely to have more severe symptoms than participants with ASD who are screened in epidemiological studies.

In our study, the SAs were more common among males in children with and without ASD. This finding differs from Ben-Sasson et al. (2009), who found no gender difference in sensory over-reactiveness. Auditory, olfactory and tactile hypersensitivity were the most common forms of SAs recognized by parents among both in children with ASD and among non-ASD children.

### Sensory Abnormalities and Autistic Traits (as Measured by the ASSQ)

The ASSQ was able to differentiate children with and without SAs in the total epidemiological child sample as well as in the non-ASD sample. This indicates that SAs have a strong impact on the behavior of a child. It is important to recognize that this is not merely an ASD-related issue, but SAs can interfere a child’s everyday life and social functioning also in the general population, and these children need help in regulating their sensory environment. According to Hazen et al. (2014), sensory over-responsivity is the most often cited sensory correlate to increased anxiety in both general and ASD populations.

In our study, among children with ASD, auditory hypersensitivity was found to be statistically significantly associated with higher QAT with a large effect size. Also among the non-ASD sample, auditory hypersensitivity was associated to higher QAT statistically significantly with an effect size of 0.03. Based on our results, auditory hypersensitivity is, thus, a notable SA that affects a child on a behavioral level in the general child population. Literature reviews and international recommendations emphasize that noise in daycare centers and schools may have injurious consequences to children’s cognitive development. Acoustics in educational settings, reverberation and group sizes have an effect on the loudness level of speech and noise (American Speech-Language-Hearing Association 1995; Evans 2006; Zuurbier et al. 2007). Therefore, auditory elements are usually taken into consideration in schools and daycare centers when planning special education, support and care for children with ASD. One possible explanation to the fact that auditory hypersensitivity had the most significant effect on child behavior in both study groups is that it can be easily perceived by caregivers since it usually leads to markable over responsivity and avoidant behavior.

Auditory hypersensitivity manifests by a discomfort or painful response to noises, for example certain types of noisy environments (Kern et al. 2001; Rosenhall et al. 1999). It is most acute if the noise level is high or if there are many different sources of noise, for example in restaurants (Kern et al. 2001). In school settings, the school cafeteria is an area where the different noises can cause problems for sensitive pupils. Noises there usually include human talk, sudden loud voices, noises from kitchen, unpleasant sounds from eating and biting, clinging of the cutlery, moving of seats and people walking around. Thus a situation that is supposed to be a relaxation between lessons can turn into a very stressful situation for a child with auditory hypersensitivity.

Auditory hypersensitivity is suggested to be a result of abnormal brain processing in children with ASD. Differences in auditory sensory processing were described by Kern et al. (2006). This observation confirms to fMRT-studies. Gomot et al. (2008) reported differences in brain activity mainly involved the right prefrontal–premotor and the left inferior parietal regions. These regions were more activated in the ASD sample than in controls when they were exposed to acoustic stimuli (Gomot et al. 2008; Rosenhall et al. 1999). Kwon et al. (2007) investigated the auditory ability of children with ASD by using auditory brainstem responses and reported that children with ASD have a dysfunction or immaturity of the central auditory nervous system. Also, abnormal cortical auditory processing was observed in children with autism measuring the regional cerebral blood flow with positron emission tomography while they were listening to speech-like sounds (Boddaert et al. 2004).

In the non-ASD sample, in addition to auditory hypersensitivity, hypersensitivity of all sensory modalities and also hyposensitivity of auditory, tactile and visual modalities were statistically significantly associated with higher QAT, although with modest effect sizes. These results suggest that SAs may manifest as autistic-like features in a child’s behavior in various ways. Tactile hypersensitivity, for example, often manifests as an avoidance of being touched or by a discomfort from wearing certain clothes (Baranek et al. 1997; Kern et al. 2001) or as a resistance to hair brushing and washing (Kern et al. 2001). In school, daycare or other social situation, tactile hypersensitivity may manifest as a general avoidance of situations or marked discomfort in situations where physical contact with other children is likely. Tactile hyposensitivity, on the other hand, may reflect as an attempt to gain tactile sensations (by touching, pushing, pumping into things on purpose) on a behavioral level.

Thus, SAs affect also children without ASD, and many children benefit from learning environments with reduced sensory stimuli. The discomfort caused by sensory overload raises the stress-level of the child which can lead to poorer adaptation and weaken the child’s ability to concentrate in the learning environment. On the other hand, children with sensory under-responsiveness need activation and change of routine to keep them engaged. More knowledge of different SAs is still needed especially among teachers in general education schools and kindergarten teachers in day care centers.

### Limitations

Limitations concerning the assessment of SAs merit note. In the present study, these abnormalities were assessed by inquiring about the presence of auditory, tactile, visual, olfactory and gustatory hyper- and hyposensitivity. That is, we did not have the possibility to use validated measures of sensory perceptual problems, because during the time of the data gathering, no validated assessment methods for sensory abnormalities were available in Finland. Also, information about QAT and SAs was derived from proxy ratings (i.e., parents evaluating the SAs and both parents and teachers evaluating the QAT). Thus, it is possible, that proxy biases effect the results, as in most studies where informant based questionnaires are used.

## Conclusions

QAT level and SAs were associated in the all three study samples and existence of SAs explained the variance in QAT (the ASSQ scores) in both ASD and non-ASD samples, indicating that SAs have a marked role in autistic-like behavior. Clinicians are reminded not only to assess SAs in children who receive an ASD diagnosis, but also among children with elevated ASSQ outcome measures.
